# Identification of a Fully Human Antibody VH Domain Targeting Anaplastic Lymphoma Kinase (ALK) with Applications in ALK-Positive Solid Tumor Immunotherapy

**DOI:** 10.3390/antib13020039

**Published:** 2024-05-07

**Authors:** Chuan Chen, Zehua Sun, Zening Wang, Seungmin Shin, Abigail Berrios, John W. Mellors, Dimiter S. Dimitrov, Wei Li

**Affiliations:** 1 Center for Antibody Therapeutics, Division of Infectious Diseases, Department of Medicine, University of Pittsburgh Medical School, Pittsburgh, PA 15261, USA; chuanc1986@gmail.com (C.C.); zes20@pitt.edu (Z.S.); ses376@pitt.edu (S.S.); jwm1@pitt.edu (J.W.M.); 2 Institute of Molecular Medicine, University of Texas Health Science Center at Houston, Houston, TX 77030, USA; zening.wang@uth.tmc.edu; 3 Department of Biological Sciences, University of Pittsburgh Kenneth P. Dietrich School of Arts and Sciences, Pittsburgh, PA 15260, USA; abigail.berrios@aboundbio.com

**Keywords:** single-domain antibodies, phage display, ALK, immunotherapy

## Abstract

The anaplastic lymphoma kinase (ALK, CD247) is a potential target for antibody-based therapy. However, no antibody-based therapeutics targeting ALK have entered clinical trials, necessitating the development of novel antibodies with unique therapeutic merits. Single-domain antibodies (sdAb) bear therapeutic advantages compared to the full-length antibody including deeper tumor penetration, cost-effective production and fast washout from normal tissues. In this study, we identified a human immunoglobulin heavy chain variable domain (VH domain) (VH20) from an in-house phage library. VH20 exhibits good developability and high specificity with no off-target binding to ~6000 human membrane proteins. VH20 efficiently bound to the glycine-rich region of ALK with an EC_50_ of 0.4 nM and a KD of 6.54 nM. Both VH20-based bispecific T cell engager (TCE) and chimeric antigen receptor T cells (CAR Ts) exhibited potent cytolytic activity to ALK-expressing tumor cells in an ALK-dependent manner. VH20 CAR Ts specifically secreted proinflammatory cytokines including IL-2, TNFα and IFNγ after incubation with ALK-positive cells. To our knowledge, this is the first reported human single-domain antibody against ALK. Our in vitro characterization data indicate that VH20 could be a promising ALK-targeting sdAb with potential applications in ALK-expressing tumors, including neuroblastoma (NBL) and non-small cell lung cancer.

## 1. Introduction

Anaplastic lymphoma kinase (ALK) is a 210 kDa tyrosine kinase (TK) receptor (CD247). It belongs to the insulin growth factor receptor super family and is highly expressed in the developing nervous system during embryogenesis, but only focally expressed in the adult brain and absent in other normal tissues [1,2]. The rearrangement and overexpression of wild-type or mutated ALK may lead to aberrant ALK signaling and cause numerous cancers [3,4,5,6,7]. These features make ALK a probable target for small-molecule inhibitor therapy and immunotherapy.

Small molecules that inhibit ALK kinase have been used for neuroblastoma (NBL) therapy. However, the kinase domain is highly prone to mutation, which could result in mutagenic escape. Limited success has been detected with small molecules [8]. To overcome the risk for mutagenic escape, targeting the ALK extracellular domain instead of the kinase domain is an alternative. Several antibodies that bind to the ALK extracellular domain showed inhibition of cancer cell growth in vitro and in vivo [9,10]. These reports demonstrated the feasibility of ALK-targeting antibody therapies. To enlist therapeutic effects, antibodies typically need to be engineered into different therapeutic modalities, including antibody–drug conjugates (ADCs), chimeric antigen receptor (CAR), T-cell and Bi-specific T-cell engagers (TCEs).

ADCs combine the advantages of the target-specific binding of antibodies and cancer-killing effects of cytotoxic drugs, thereby discriminating healthy and cancer cells during therapy [11,12]. Currently, several ADCs have been approved by the FDA for oncology [13]. ADC targeting ALK demonstrates high killing efficacy in preclinical models of NBL [14]. CAR T cell immunotherapy has revolutionized therapy for blood tumors, especially for B cell malignancies [15,16,17,18,19], although many hurdles exist for applications in solid tumors [20,21,22]. ALK-targeting CAR Ts have shown effective antitumor efficacy [23]. TCEs are fusion proteins consisting of a CD3 antibody and an antigen-specific antibody. TCEs can effectively mobilize T cells to kill tumor cells, as evidenced by a plethora of in vitro and in vivo studies [24]. Currently, several TCEs have been approved by the FDA, with hundreds of TCEs being evaluated in preclinical and clinical trials [25].

Currently, all the reported ALK antibodies are full-size antibodies with paired heavy chains and light chains. Recently, sdAbs as targeting moieties have become attractive due to their unique therapeutic merits, such as targeting occluded epitopes, deep tumor penetration and high expression level and stability, etc. Here, we identified a fully human antibody VH domain (VH20) that targets human ALK with high specificity and affinity. Based on the VH20, we constructed TCE and CAR T as cancer immunotherapeutics. Both TCE and CAR Ts showed specific and efficient killing against 293T-ALK cells and NBL cells (SY5Y and IMR32) that intrinsically express ALK, without cytotoxicity against ALK-negative SK-N-AS cells. Our results highlight the development of a novel fully human sdAb targeting ALK, and in vitro function of TCEs and CAR Ts, which may be good candidate immunotherapeutics for ALK-expressing tumors.

## 2. Materials and Methods

### 2.1. Cell Culture

293T (ATCC, CAT#CRL-3216) and 293T-ALK stable cells were cultured in the DMEM medium supplemented with 10% fetal bovine serum (FBS, HyClone, Fisher Scientific, Waltham, MA, USA) and maintained at 37 °C in an incubator with 5% CO_2_. The original Expi293F (ThermoFisher, CAT#A14527, New York, NY, USA) cells and all the stable cell pools generated based on Expi293F cells were cultured in Expi293 Expression Medium according to the manufacture’s protocol. Then, 100 μg/mL Zeocin antibiotic was added into the stable cell pools to maintain the expression of the proteins. PanT (STEMCELL Technologies, CAT#70024, Cambridge, MA, USA) and VH20 CART cells were maintained in TexMACS medium containing 50 IU/mL IL-2 (Miltenyi Biotec, Auburn, AL, USA). SH-SY5Y (ATCC, CAT#CRL-2266, Manassas, VA, USA) cells were cultured in an EMEM and F12 1:1 mixture medium supplemented with 10% FBS at 37 °C in an incubator with 5% CO_2_. IMR-32 (ATCC, CAT#CCL-127) cells were cultured in EMEM medium with 10% FBS in an 2.5% CO_2_ incubator at 37 °C. DMEM medium supplemented with 10% FBS and 0.1 mM Non-Essential Amino Acids (NEAA) was used to maintain the SK-N-AS (ATCC, CAT# CRL-2137) cells at 37 °C in an incubator with 5% CO_2_.

### 2.2. Plasmid Construction

**Figure 1:**
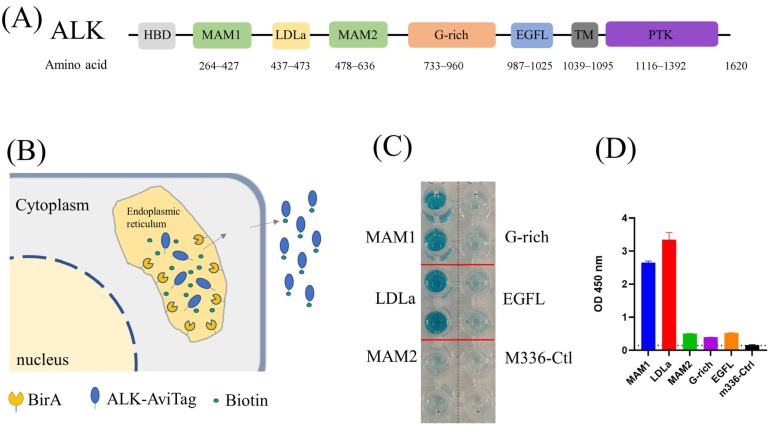
(**A**) Architecture of ALK. (**B**) Schematic of protein biotinylation in the BirA enzyme expression cell. The protein was biotinylated in the endoplasmic reticulum (ER) by ER–retained biotin ligase (BirA-ER). After folding and modification, the biotinylated protein was then secreted into the medium. (**C**) ELISA results of biotin-labeled ALK antigen. Compared with M336 control, all purified ALK domains were successfully biotinylated. (**D**) Quantification of ELISA results from (**C**).

For antigen expression, a secretory signal peptide was added into the N-terminal of the ALK MAM1 (AA263-430), LDL (AA434-477), MAM2 (AA478-640), G-rich (AA641-1038), EGFL (AA985-1025) and ALK extracellular (AA19-1038) domains by PCR, and the sequences (Figure 1A) were next cloned into our in-house pZeo-ATHis plasmid between *HindIII* and *EcoRI* to generate the pZeo-MAM1, pZeo-MAM2, pZeo-LDL, pZeo-G-rich, pZeo-EGFL and pZeo-ALK ecto expression vectors. A IgG1 Fc fragment was then cloned into pZeo-ALK ecto plasmid between *StuI* and *EcoRI* to generate the pZeo-ALK ecto-Fc vector. For ALK display, the PDGFR transmembrane domain (TM) was cloned into pZeo-ALK ecto plasmid between *StuI* and *EcoRI* to generate the pZeo-ALK-TM vector. For VH20-TCE expression, the VH20 and OKT3-7 scFv genes were fused by overlap-PCR and cloned into pZeo-Fc between *HindIII* and *BspEI* to generate pZeo-VH20-OKT3-Fc. For CAR T experiments, the VH20 gene with a secretory signal peptide and a Flag-tag was cloned into pSLCAR plasmid between *BamHI* and *KpnI* to generate the pSLCAR-VH20 vector.

### 2.3. Transfection and Stable Cell Pool Establishment

293T cells were used for 293T-ALK stable cell pool selection. Approximately 24 h before transfection, 2 × 10^5^ cells were seeded into a 35 mm dish. The next day, 2 μg pZeo-ALK-TM vector was mixed with 8 μg polyethyleneimine (PEI) HCl MAX (MW = 40,000, Polysciences) in 200 μL Opti-MEM medium (ThermoFisher, Waltham, MA, USA). After 20 min incubation, the DNA/PEI mixtures were added into 293T cells and the cells were maintained at 37 °C in an incubator with 5% CO_2_ for 24 h. Next, the cells were transferred into a T75 flask and the stable cell pool was selected with 500 µg/mL Zeocin in the cell culture medium for 7 days. The live cells were the 293T-ALK stable cells with the ALK extracellular domain (ALK ecot) displayed on the cell surface.

For biotin-labeled ALK domains’ expression, the pZeo-MAM1, pZeo-MAM2, pZeo-LDL, pZeo-G-rich, pZeo-EGFL and pZeo-ALK ecto vectors were transfected into our in-house BriA enzyme expression Expi293F cell line (Expi293F-BriA). For ALK-Fc and VH20-TCE expression, pZeo-ALK ecto-Fc and pZeo-VH20-OKT3-Fc plasmid were transfected into Expi293F cell line. The stable cell pool selection process followed the description in our previously published manuscript [26].

### 2.4. Protein Expression and Purification

The selected stable cell pools were used for protein expression. The diluted cells were adjusted with Expi293 expression medium to the concentration of 2 × 10^6^ cells/mL in round bottles (for biotin-labeled proteins, 100 μM biotin was added into the medium), and maintained at 37 °C with shaking at the speed of 135 rpm in an 8% CO_2_ incubator for 6 days. Then, the culture supernatant was clarified by centrifugation, the ALK-Fc and VH20-OKT-Fc were purified with protein A affinity chromatography (GenScript). Biotin-labeled MAM1, LDLa, MAM2, G-rich and EGFL domains, which contain a 6xHis Tag, were purified with Ni-NTA resin (Thermo Fisher Scientific). The productivity of biotin-labeled MAM2, G-rich and EGFL domains was extremely low (~50 μg/L). The protein A and Ni-NTA resin purification process was carried out according to the manufacturer’s protocol. Spectrophotometry (NanoVue, GE Healthcare, Chicago, IL, USA) and SDS-PAGE was used to measure the concentration and purify of the proteins.

### 2.5. ELISA

ELISA was used to confirm the success of protein biotinylation. In addition, 0.25 μg/well of purified biotin-labeled MAM1, LDLa, MAM2, G-rich and EGFL domains in 50 µL DPBS was added into ninety-six-well ELISA plates (Corning 3690) and incubated at 4 °C overnight. The next day, fresh 5% nonfat milk (Bio-RAD, Hercules, CA, USA, CAT# 1706404) in DPBS (5% PBS-Milk) was prepared. The plates were blocked at 37 °C for two hours with 150 µL 5% PBS-Milk. After blocking, the plates were washed with PBST (0.05% Tween 20 in DPBS) 3 times with a plate washer (BioTek, Winsooki, VT, USA). Then, 50 µL of 1:5000 DPBS-diluted streptavidin-HRP (Thermo) was added into each well, followed by incubation for 45 min at room temperature. The plates were washed with PBST 5 times. Then, 50 µL TMB substrate (Sigma) was added into each well; after a 2–10 min reaction, 50 µL H_2_SO_4_ (1M, Sigma, Allentown, PA, USA) was used to stop the reaction and the 450 nm absorbance was recorded with a microplate reader. The results were analyzed with GraphPad Prism 9.0.2 (Boston, MA, USA).

Furthermore, 0.25 μg/well ALK ecto protein or BSA in 50 µL DPBS was added into ninety-six-well ELISA plates (Corning 3690) and incubated at 4 °C overnight. The next day, fresh 5% nonfat milk (Bio-RAD, CAT# 1706404) in DPBS (5% PBS-Milk) was prepared and used for blocking the plates at 37 °C for two hours. After blocking, the plates were washed with PBST (0.05% Tween 20 in DPBS) 3 times with a plate washer (BioTek). Then, the 3-fold serially diluted VH20, and 5-fold serially diluted VH20-OKT-Fc in the presence of 5% PBS-Milk were added in a 50 μL volume. Diluted antibodies were incubated for 1 h at room temperature. Then, the plates were washed with PBST 4 times. Next, the 1:1000 diluted anti-Flag-HRP (ThermoFisher, Waltham, MA, USA) or Mouse anti-Human IgG-HRP (Thermo, CAT# 05-4220) in 5% PBS-milk were added into each well and incubated for 1 h at room temperature. After washing with PBST for 5 times, 50 µL TMB substrate (Sigma) was added into each well; after a 2–10 min reaction, 50 µL H_2_SO_4_ (1M, Sigma) was used to stop the reaction and the 450 nm absorbance was measured with a microplate reader (BioTek, Winsooki, VT, USA). The results were analyzed with GraphPad Prism 9.0.2.

### 2.6. Flow Cytometry

293T, 293T-ALK, SK-N-AS, IMR-32, SH-SY5Y and human peripheral blood PanT cells were used to measure VH20 and VH20-TCE binding to cell-surface-associated ALK and CD3 by FACS. Briefly, ~10^6^ cells were stained with VH20 and VH20-TCE antibodies at a concentration of 500 nM and 100 nM in 200 µL PBS on ice for 30 min. Then, the cells were washed three times with 1 mL PBS. Meanwhile, 200-fold-diluted anti-Flag-PE antibody (BioLegend, San Diego, CA, USA) and goat anti-human IgG (γ-chain specific)-R-PE (Sigma) were prepared in PBS. The cells were then stained with 200 μL diluted second antibodies on ice for 30 min. After washing three times with 1 mL PBS, the cells were detected with a BD LSR II (San Jose, CA, USA).

Furthermore, ~10^6^ PanT and VH20 CAR T cells were used to measure the VH20 CAR-recombinant lentiviruses’ transduction efficiency by detecting GFP positive cells on a BD LSR II. All FACS data analyses were performed using FlowJo_V10. (BD Biosciences, San Jose, CA, USA)

### 2.7. Dynamic Light Scattering (DLS)

We changed the buffer of VH20 with DPBS and adjusted the concentration to 2 mg/mL. Then, the VH20 solution was filtered with a 0.22 μM filter and incubated at 37 °C for DLS. The size distributions of protein particles were determined with Zetasizer Nano-ZS ZEN 3600 (Malvern Instruments Limited, Westborough, MA, USA). The samples were measured at day 0, day 1, day 3 and day 7 and Day 14.

### 2.8. Size Exclusion Chromatography (SEC)

Briefly, 200 µL (1 mg/mL) filtered VH20 was injected into the Superdex 200 Increase 10/300 GL chromatography (GE Healthcare, Cat. No. 28990944) column and an analysis was performed with DPBS at a flow rate of 0.4 mL/min. Protein sets of Ferritin (440 kDa), Aldolase (158 kDa), Conalbumin (75 kDa), Ovalbumin (44 kDa), Carbonic anhydrase (29 kDa), and Ribonuclease A (13.7 kDa) were used to calibrate the column.

### 2.9. VH20-TCE Cytotoxicity Assay

The PanT cells (STEMCELL, CAT#70024) were activated with anti-CD3/CD28 beads (Thermo, CAT#11131D) for 24 h. Then, the beads were removed and the cells were maintained in a fresh medium for 4 days until their viability was >70%. Furthermore, 10,000 cells/well target tumor cells were mixed with either activated or non-activated T cells at the effector to target (E/T) ratio of 5:1 in 96-well white plates (Corning) in 100 μL of culture medium. Then, 100 μL serially diluted VH20-TCE was added into the cell mixture and maintained at 37 °C in an incubator with 5% CO_2_ for 24 h. Cytotoxicity was determined by the LDH-Glo^TM^ Cytotoxicity Assay (Promega, CAT#J2380, Madison, WI, USA) according to the manufacturer’s protocol. The percentage of cytotoxicity was calculated with the following formula: Cytotoxicity (%) = (Experimental lysis − Effector spontaneous lysis − Target spontaneous lysis)/(Target maximum lysis − Target spontaneous lysis) × 100%. The curves were analyzed with GraphPad Prism 9.

### 2.10. VH20 CAR T Cell Generation

The VH20 CAR lentivirus were packaged by co-transfecting Lenti-X^TM^ 293T cells (Fisher Scientific, CAT# NC9834960, Waltham, MA, USA) with recombinant plasmids pSLCAR-VH20, pSPAX2 and pMD2.G. Lentivirus packaging, concentration and purification were performed based on the Lentivirus Production Protocol (Addgene, Watertown, MA, USA).

PanT cells (STEMCELL # 200-0046) were used for the VH20 CAR in vitro cytotoxicity assay. PanT cells were activated with anti-CD3/CD28 beads for 24 h. The next day, the activated T cells were mixed with VH20 CAR lentiviruses at a multiplicity of infection (MOI) value of 20. Then, ~8 µg/mL polybrene was added to enhance the transduction efficiency. We then centrifuged the plates at 800× *g* for 1.5 h at 32 °C and incubated them for 12 h in 37 °C cell culture incubator. The transduced cells were then transferred into a bigger flask, followed by the addition of a fresh medium, and the cells were cultured for 6 days. We then removed the anti-CD3/CD28 beads and culture cells for another 7 days. The VH20 CAR T cells’ transduction efficacy ranged from 85% to 89.3% due to experimental variations.

### 2.11. In Vitro VH20 CAR T Cytotoxicity Assay

VH20 CAR T cytotoxicity was tested against 293T, 293T-PRTG, SK-N-AS, IMR-32 and SH-SY5Y target cells. PanT cells (as controls) and VH20 CAR-T cells (effector cells) were incubated with target cells (10,000 cells/well) at different E/T ratios of 1:1, 2:1, 4:1, 8:1 and 16:1 for 48 h in 96-well cell culture plates (Corning). Cytotoxicity was determined with LDH-Glo™ Cytotoxicity Assay (Promega, CAT#J2380) according to the manufacturer’s protocol. The percentage of cytotoxicity was calculated with the following formula: Cytotoxicity (%) = (Experimental lysis − Effector spontaneous lysis – Target spontaneous lysis)/(Target maximum lysis − Target spontaneous lysis) × 100%. The curves were analyzed with GraphPad Prism 9.

### 2.12. Cytokine Release Assay

After 48 h of co-culture of target cells and effector cells, supernatants were harvested for the detection of cytokines’ (IL2, IFN-r and TNFa) release using the interleukin-2 Human ELISA Kit (Thermo, CAT# 88-7025-22), IFN gamma Human ELISA Kit (Thermo, CAT# 88-7316-22) and TNF alpha Human ELISA Kit (Thermo, CAT#88-7346-22), which were used according to the manufacturer’s protocols. The curves were analyzed with GraphPad Prism 9.

## 3. Results

### 3.1. Production of Biotin-Labeled ALK Domains for Phage Panning

ALK is a single transmembrane protein with extracellular, transmembrane and intracellular domains (Figure 1A). The ectodomain is further divided into several subdomains, including MAM, G-rich region and EGFL domains with distinct functions [27]. To identify the ALK-specific antibody, we first utilized the recombinant full-length ALK ectodomain protein (ALK-ecto) to pan against our in-house proprietary antibody phage libraries [28]. Screening ELISA output dozens of positive clones. However, none of them bound with cell-surface-associated ALK, indicating the conformational difference between heterologous produced recombinant ALK protein and cell-surface-associated ALK. We then truncated the large ALK ectodomain into subdomains (MAM1, LDLa, MAM2, G-rich and EGFL) and expressed them individually in expi293F cells. Meanwhile, we utilized an in vivo biotinylation strategy to label those subdomains [29], in which a BriA enzyme coding gene is co-transfected with ALK subdomains that are engineered to contain a C-terminal Avitag (Figure 1B). During protein expression, the BirA enzyme and our target proteins with AviTag were transferred into endoplasmic reticulum (ER). The BirA enzyme was retained on the ER catalyzes biotin specifically attaching to the lysine residue of AviTag (GLNDIFEAQKIEWHE). The biotin-labeled antigens were purified by Ni-NTA (SDS-PAGE) and their biotinylation efficacy was confirmed by ELISA (Figure 1C,D).

### 3.2. Bio-Panning against Phage-Antibody Libraries Using ALK Subdomains Outputs VH20 with High Binding Affinity and High Specificity

**Figure 2:**
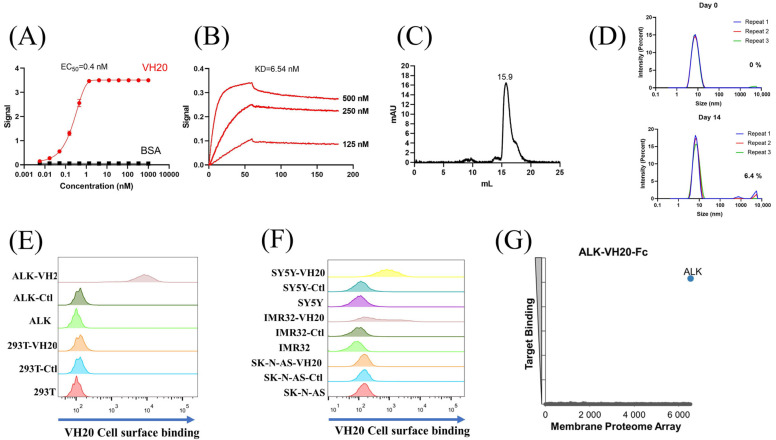
Characterization of ALK sdAb VH20. (**A**) ELISA results of VH20 binding to ALK extracellular domain. The BSA was used as a negative control. ELISA samples were tested in triplicate and error bars denote ± SD, *n* = 3. (**B**) BLItz result of VH20 binding to ALK-Fc. (**C**) Aggregation evaluation of VH20 by size exclusion chromatography (SEC). (**D**) Aggregation evaluation of VH20 by dynamic light scattering (DLS). Antibody was evaluated with a concentration of 2 mg/mL. 6.4% VH20 aggregation was detected after 14 days of incubation at 37 °C. (**E**) FACS results of VH20 binding to 293T and 293T-ALK cells. VH20 specifically binding to 293T-ALK cells, with none off-target binding to 293T cells. (**F**) FACS results of VH20 binding to NBL SK-N-AS, IMR-32 and SH-SY5Y cells. VH20 specifically binds to IMR-32, SH-SY5Y cells with no binding to SK-N-AS cells. (**G**) Membrane Proteome Array (MPA) result of VH20-Fc (20 μg/mL) against different human membrane proteins (>6000) and no non-specific binding was detected.

After three rounds of panning using different biotin-labeled subdomains against our in-house fully human VH phage-display libraries [28], a dominant clone, VH20 was enriched with the G-rich domain. VH20 bound to the ALK-ecto as measured by ELISA with a half-maximal binding concentration (EC_50_) of 0.4 nM (Figure 2A). Biolayer interferometry (Blitz) showed potent binding of VH20 to ALK-Fc (ALK-ecto fusion with human IgG1 Fc) with an equilibrium dissociation constant (K_D_) of 6.54 nM (Figure 2B). The size exclusion chromatography (SEC) results indicated that VH20 exhibits a homogenous folding status (Figure 2C). Dynamic light scattering (DLS) showed that VH20 was stable and aggregation-resistant at 37 °C. Less than 7% aggregation was detected after 14 days of incubation (Figure 2D).

Having tested VH20’s antigen binding and its biophysical properties, we sought to evaluate VH20 binding to cell-surface-associated ALK by flow cytometry. Firstly, we generated an isogenic 293T cell lines that stably express ALK (293-ALK). As Figure 2E shows, VH20 specifically bound to 293T-ALK cells, but without any binding to ALK-negative 293T cells. Next, we evaluated VH20 binding to three different NBL cell lines (IMR-32, SH-SY5Y ALK-positive cells, and SK-N-AS ALK-negative cells). The results showed that VH20 specifically binds to ALK-positive IMR-32 and SH-SY5Y cells, but no binding to ALK-negative SK-N-AS ALK cells was observed (Figure 2F).

Antibody specificity is critical for its clinical therapeutic application. We further confirmed the specificity of VH20 by the Membrane Proteome Array Assay (MPA) [30,31,32,33], in which the cross-reactivity of VH20 to a total of 6000 distinct human membrane proteins containing more than 94% of the human membrane proteome (including transporters, ion channels, GPCRs, etc.) was tested in high-throughput flow cytometry. The results showed that VH20 only bound to cell-membrane-associated ALK, without any binding to other 6000 human membrane proteome (Figure 2G).

These results demonstrated the specificity and strong binding affinity of our first-ever identified ALK-targeting fully human antibody VH domain (VH20), which lay the ground for its cancer immunotherapeutic applications.

### 3.3. Design and Generation of VH20-Based T Cell Engager (TCE) for Cancer Immunotherapy

**Figure 3:**
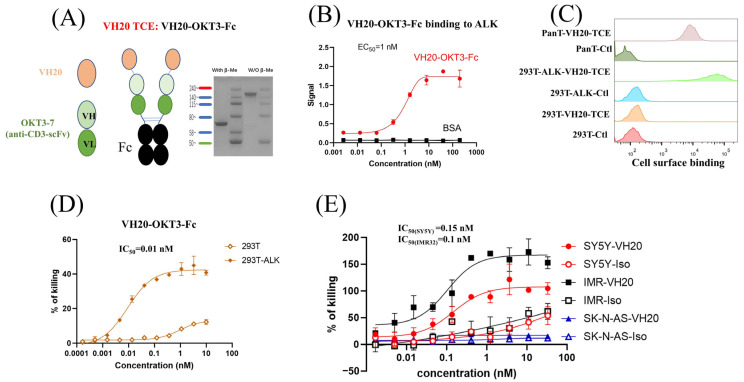
VH20-TCE (VH20-OKT-Fc) structure and T cell-mediated cytotoxicity. (**A**) The scheme of the VH20-TCE. VH20-TCE consists of VH20 fusing at the N terminus of OKT3 scFv followed by linking to the human IgG1 Fc. (**B**) ELISA results of VH20-TCE binding to ALK extracellular domain with BSA as a negative control. Samples were tested in triplicate and error bars denote ± SD, *n* = 3. (**C**) FACS results of VH20-TCE binding to 293T, 293T-ALK and PanT cells. VH20-TCE specifically binds to 293T-ALK and PanT cells. No off–target binding to 293T cells was detected. (**D**) Cytotoxicity of T cells (activated) to 293T and 293T-ALK cells induced by VH20-TCE. Potent killing efficacy towards 293T-ALK cells was detected. (**E**) Cytotoxicity of T cells to IMR−32, SH-SY5Y and SK-N-AS cells induced by VH20-TCE. Potent killing efficacy towards IMR-32 and SH-SY5Y cells was detected. TCE cytotoxicity assays were detected in triplicate and error bars denote ± SD, *n* = 3.

As a proof-of-concept study, a bispecific T cell engager was constructed by linearly fusing VH20 with the widely used humanized anti-human CD3 antibody single-chain variable fragment (OKT3-7-scFv) [34]. In order to enhance the binding activity and extend the antibody’s half-life, the tandem VH20-OKT3-7-scFv was fused with the human IgG1 Fc domain to generate VH20-OKT3-Fc (Figure 3A). VH20-OKT3-Fc was recombinantly expressed in Expi293 cells, and the purity of one-step protein A purification reached 95%, as revealed by SDS-PAGE (Figure 3A). VH20-OKT3-Fc specifically bound to the recombinant ALK-eco protein with an EC_50_ of ~1 nM without any binding to BSA (Figure 3B). In addition, VH20-OKT3-Fc retained binding to cell-surface-associated ALK on 293T-ALK cells. Its binding was specific, as no binding was observed in 293T cells. Moreover, VH20-OKT3-Fc bound to cell surface CD3 on PanT cells (Figure 3C).

We then tested VH20-OKT3-Fc-mediated T cell cytotoxicity by using multiple ALK-overexpressing cell lines. We firstly used the ALK-293T cells as target cells. The CD3/CD28 beads activated human PanT cells enriched by CD3+ positive selection from PBMCs of healthy donors were used as effector cells. We observed that VH20-OKT3-Fc-induced T cells’ killing of 293T-ALK cells was concentration-dependent with an EC_50_ of 0.01 nM (Figure 3D). The killing of 293T-ALK is significantly higher than that of ALK-negative 293T cells, demonstrating the specificity of VH20-OKT3-Fc. Nevertheless, we observed a marginal killing of 293T cells at a high concentration of VH20-OKT3-Fc, which may be because of the over-activation of PanT cells, leading to non-specific killing. Furthermore, we evaluated VH20-OKT3-Fc mobilized T cell cytolysis against NBL cells, including ALK-positive cells IMR-32, SH-SY5Y and ALK-negative SK-N-AS cell line. The results showed that VH20-OKT3-Fc mobilized the T cell killing of ALK-positive IMR-32 and SH-SY5Y cells with IC_50_s of 0.1 nM and 0.15 nM, respectively, while without killing of ALKnegative SK-N-AS cancer cell. In addition, VH20-OKT3-Fc induced T cell killing appears specific as its cytotoxicity was much higher than that induced by the isotype control, which has the same molecular architecture as VH20-OKT3-Fc except when using an irrelevant VH to replace VH20 (Figure 3E).

**Figure 4:**
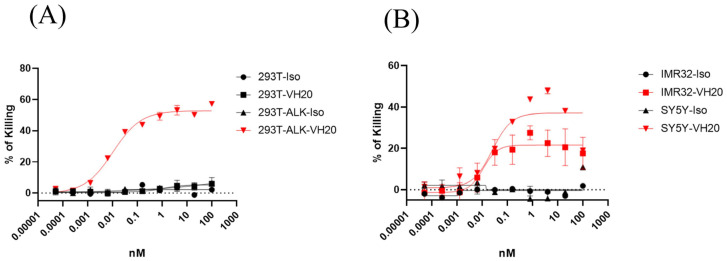
Cytotoxicity of non-activated T cells to 293T, 293T-ALK (**A**) and IMR-32, SH-SY5Y. (**B**) induced by VH20-TCE. Potent killing efficacy towards IMR-32 and SH-SY5Y cells were detected. TCE cytotoxicity assays were tested in triplicate and error bars denote ± SD, *n* = 2.

We also tested the mobilization of unactivated T cells for cell killing mediated by VH20-OKT3-Fc (Figure 4). The results showed similar killing EC_50_ on 293T-ALK, IMR-32 and SH-SY5Y cells compared to the activated T cells. However, the killing of non-activated T cells seems more specific since we barely observed cell killing for the isotype control TCE or for the VH20-TCE against ALK-negative 293T cells. In addition, as expected, the killing efficacy (maximum killing percentage) for non-activated T cells is lower than that of activated T cells against SH-SY5Y and IMR-32 cells.

Taken together, these T cytotoxicity efficacy results suggest the potential of our ALK-targeting VH20 to be used in the context of bispecific T cell engager for the eradication of ALK-positive tumor cells. 

### 3.4. Development of VH20-Based Chimeric Antigen Receptor (CAR) T Cells for Killing of ALK-Positive Cancer Cells 

**Figure 5:**
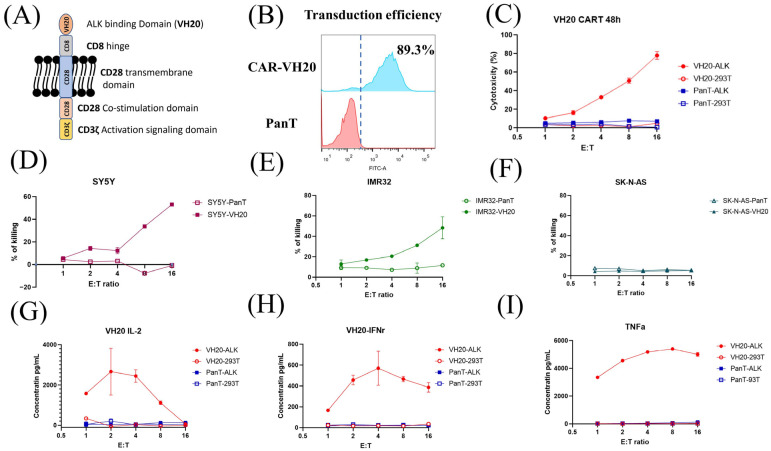
VH20-CAR structure scheme and CAR T cell-mediated cytotoxicity and cytokine release. (**A**) The structure of VH20 CAR. VH20, CD8 hinge, CD28 transmembrane domain, CD28 co–stimulation domain and CD3z activation signal domain were used. (**B**) VH20 CAR T cells’ transduction efficiency. The GFP fluorescent was detected with FACS to evaluate transduction efficiency, ~89.3% of T cells were detected GFP–positive. (**C**) Killing efficacy of VH20 CAR T cells towards 293T and 293T-ALK cells. PanT cells were used as control. (**D**–**F**) Killing efficacy of VH20 CAR T cells towards SH-SY5Y (**D**), IMR-32 (**E**) and SK-N-AS (**F**) cells. Significant killing of IMR-32 and SH-SY5Y cells was detected with high E/T ratio. No killing to SK-N-AS cells was detected. VH20 CAR T cytotoxicity assay was carried out in triplicate and error bars denote ± SD, *n* = 3. (**G**–**I**) Cytokine release was measured by ELISA. Significant IL-2 (**G**), INFr (**H**) and TNFa (**I**) expression were detected. Cytokine release assay was carried out in triplicate and error bars denote ± SD, *n* = 3.

Next, we tested how VH20 works in CAR T cells. To generate VH20 CAR, the VH20 antibody gene was cloned into a lentiviral CAR plasmid (pSLCAR), which harbors a cassette including the CD8 extracellular hinge region, the CD28 transmembrane domain and CD28 intracellular stimulatory domain, followed by the CD3 zeta domain (Figure 5A) [35]. The VH20 CAR plasmid and the package plasmids pMD2.G and pSPAX2 were co-transfected into lenti-293 cells for the production of lentivirus. For transduction, the PanT cells enriched from hPBMCs were activated using CD3/CD28 dynabeads and subsequently transduced with VH20-CAR lentivirus with a MOI of 20 using 8 µg/mL polybrene to enhance the transduction efficiency. After expansion, the PanT cell transduction efficiency was tested by flow cytometry, with around 89.3% positively transduced cells (Figure 5B).

The cytotoxicity of VH20-CAR T cells was measured on 293T-ALK cells. The results showed that VH20-CAR Ts induced cytotoxicity of 293T-ALK cells at a E/T ratio as low as 1:1. VH20-CAR Ts eliminated the majority (78.1%) of 293T-ALK cells at a E/T ratio of 16:1 after 48 h of co-culture (Figure 5C). VH20-CAR T cells’ killing is specific, as no cytotoxicity was observed on ALK-negative 293T cells at any E/T ratios. Furthermore, we tested the VH20-CAR Ts’ killing of NBL cells (Figure 5D–F). The results showed that VH20-CAR Ts killed ALK-positive SH-SY5Y and IMR-32 cells in a E/T ratio-dependent manner. At a ratio of 16:1, VH20-CAR Ts eradicated 48.3% and 53% of IMR-32 and SH-SY5Y cells, respectively. The untransduced PanT cells did not show any toxicity towards ALK-positive IMR-32 and SH-SY5Y cells. Meanwhile, VH20-CAR Ts did not show cytotoxicity for the ALK-negative SK-N-AS cells, further supporting the specificity of VH20-CAR Ts. 

We then sought to detect the cytokine release of VH20-CAR Ts after incubation with ALK-positive cells. The results showed that VH20-CAR Ts secreted proinflammatory cytokines, including interleukin-2 (IL-2), Interferon-gamma (IFN-γ), and tumor necrosis factor alpha (TNF-α) in an ALK-expression and E/T-dependent manner (Figure 5G–I). Interestingly, at high E/T ratios of 8:1 and 16:1, CAR Ts cytokine secretion levels became lower than those at a low E/T ratio, which may be due to the over-killing of 293T-ALK cells by CAR Ts at a high E/T ratio, leading to less antigen stimulation of CAR Ts. 

## 4. Discussion

ALK is a lineage-restricted oncoprotein expressed on the cell surface of NBL as wild-type (WT), mutated, or amplified kinase, and recently shown to be overexpressed in fusion-positive rhabdomyosarcoma (RMS) [36]. Clinically, small-molecule ALK kinase inhibitors have shown potent therapeutic efficacy for NBL with ALK mutations or amplifications [37,38]. But these molecules are limited to a minority of the high-risk population, and resistance resulting from mutations in the highly mutated kinase domain was detected [39,40]. To overcome the drawbacks of small molecules, some groups have tested ALK-specific antibodies to interfere with the ALK downstream signaling pathway for ALK-positive cancer therapy [9,10,41,42]. On the other hand, since ALK expression is restricted to tumor tissues with minimal expression in normal tissues, ALK is a potential target for NBL immunotherapy. Recently, ALK-targeting CAR T and ADC have been developed, showing potent tumoricidal efficacy in vitro [23,43]. Previously identified ALK binders are full-size antibodies. Here, we identified an ALK-specific fully human antibody VH domain (VH20). VH20 exhibits good drug ability and developability, as evidenced by its homogenous folding and aggregation resistance. More importantly, VH20 showed high specificity, as revealed by the human membrane proteome binding assay, in which VH20 did not cross-react with ~6000 human surface proteins. These properties, combined with VH20’s high binding affinity, make VH20 a strong candidate for ALK-targeting immunotherapeutics, including bispecific immune cell engagers, CARs, and ADCs.

As proof-of-concept studies, we generated VH20-based T cell engager (TCE) and CAR Ts. Both VH20-TCE and CAR Ts exhibit specific killing of target cells in an ALK-dependent manner. TCE and CAR Ts are able to take effect both on ALK transgenic 293T cells (293T-ALK) and cancer cells that intrinsically express ALK. Intriguingly, while SH-SY5Y carries mutant ALK, IMR-32 harbors WT non-amplified ALK [44]. The fact that VH20 targeting TCE and CARs eradicates both cell lines indicates that VH20 targeting ALK seems independent of ALK mutation status, which might be superior to traditional TKIs and can be applicable to a broad population of cancer patients. We also observed that VH20-TCE can induce T cytotoxicity against cancer cells when T cells are both pre-activated and non-activated (Figure 3 and Figure 4). The recruitment and mobilization of “cold” T cells by TCEs is a desirable property, which can potentially ignite and recruit TILs in immuno-“cold” tumors.

In summary, a fully human, first-ever antibody VH domain against ALK with high binding affinity and high specificity was identified. The T-cell-engager and CAR T-based VH20 exhibited potent and specific tumoricidal activity in vitro. Its efficacy in vivo warrants further investigation in preclinical animal models. In addition, VH20 can also be used as a delivery carrier for ALK-targeting bioimaging (immunoPET), radiotherapy (RPT), and chemotherapy (ADC).

## Data Availability

All data supporting the findings of this study are available within this paper and are available from the corresponding author upon request.
